# Pericardioesophageal fistula

**DOI:** 10.1002/ccr3.4844

**Published:** 2021-12-09

**Authors:** Amr Mohamed

**Affiliations:** ^1^ Department of Internal Medicine Rochester General Hospital Rochester New York USA

**Keywords:** chest pain, dysphagia, fistula, pneumopericardium

## Abstract

Late‐onset chest pain, dysphagia, or endocarditis‐like symptoms after atrial fibrillation ablation could be an alarm for fatal complications as Esophagoatrial or Pericardioesophageal fistula. If the diagnosis is delayed, mortality will be inevitable.

## Graphical abstract

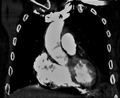

Chest pain immediately after atrial fibrillation ablation is seen essentially with every case, and we counsel the patients that it is expected. On the other hand, delayed chest pain is not always expected and usually points towards a procedure complication, as shown in this case.

This is a 70‐year‐old woman with a past medical history of atrial fibrillation status post atrial fibrillation ablation procedure 2 weeks ago. She is now presenting with pericardial chest pain and dysphagia, 2 weeks after the ablation procedure. Her vital signs are normal, and her physical examination is unrevealing.

Computerized Tomography (CT) scan of the chest showed pneumopericardium with evidence of pericardioesophageal fistula. She underwent pericardial window and esophageal stenting, and her postoperative course was uneventful.

From this case, the critical clinical message is to know that Late‐onset chest pain, dysphagia, or endocarditis‐like symptoms post atrial fibrillation ablation could be an alarm for fatal complications as Esophagoatrial or Pericardioesophageal fistula.^1^ If the diagnosis is delayed, mortality will be inevitable. Having a high index of suspicion is the key to early diagnosis.

## CONFLICTS OF INTEREST

None.

## AUTHOR CONTRIBUTIONS

The author was soley responsible for data collection, analysis and presentation.

## ETHICAL APPROVAL

Patient consent had been obtained for the publication of the study material.

## Data Availability

The data that support the findings of this study are available from the corresponding author upon reasonable request.
